# Quantitative Detection and Follow-Up of Intracranial Hypertension in Craniosynostosis: An Optical Coherence Tomography Study

**DOI:** 10.1097/PRS.0000000000011177

**Published:** 2023-11-06

**Authors:** Bianca K. den Ottelander, Stephanie D. C. van de Beeten, Sumin Yang, M. L. C. van Veelen, Robert C. Tasker, Sjoukje E. Loudon, Irene M. J. Mathijssen

**Affiliations:** Rotterdam, the Netherlands; and Boston, MA; From the 1 Department of Plastic and Reconstructive Surgery and Hand Surgery, Dutch Craniofacial Center; 2 Department of Neurosurgery; 4 Department of Ophthalmology, Erasmus Medical Center–Sophia Children’s Hospital, University Medical Center Rotterdam; 3 Department of Anesthesia (Pediatrics), Harvard Medical School and Boston Children’s Hospital.

## Abstract

**Background:**

In patients with craniosynostosis, the authors evaluated the diagnostic accuracy of fundoscopy and optical coherence tomography (OCT) to detect intracranial hypertension (ICH), the time course of retinal thickness after treatment of ICH, and the relationship between high hyperopia (HH) and fundoscopy/OCT scan findings.

**Methods:**

Patients with syndromic, multisuture, unicoronal, unilambdoid, or sagittal synostosis visiting the authors’ national center were included in this longitudinal cohort study and formed a consecutive series. Retinal layers on OCT, OCT fundus images, and fundoscopy results were evaluated. ICH was scored according to presence of abnormal intracranial pressures, hydrocephalus, progressive cerebellar tonsillar herniation or fingerprinting, and growth arrest. Diagnostic accuracy of OCT, fundoscopy, and fundus image; the time course of retinal thickness after ICH; and interference of HH were analyzed using linear mixed models.

**Results:**

A total of 577 OCT scans in 307 patients were included. ICH was found in 7.2%. Combining total retinal thickness (TRT), OCT fundus imaging and fundoscopy resulted in a sensitivity of 76% and 81% specificity to detect signs of ICH. TRT was increased in patients who had had signs of ICH versus patients who had never had signs of ICH (β +44.9 µm in patients who had had ICH [95% CI, 9.0 to 80.8]; *P* = 0.01). TRT decreased to normal in the years after surgery (β −3.6 µm/yr [95% CI, −7.2 to −0.05]; *P* = 0.047). There were greater odds of having increased TRT in patients with HH (OR, 2.9 [95% CI, 1.1 to 7.6]; *P* = 0.03).

**Conclusions:**

The correlation among TRT, OCT fundus image, fundoscopy, and particularly the combination of these measures with intracranial pressure surrogate markers is fair. Increased TRT in the presence of a clinical suspicion of ICH warrants further screening.

**CLINICAL QUESTION/LEVEL OF EVIDENCE:**

Diagnostic, III.

Detecting intracranial hypertension (ICH) is one of the main challenges of managing patients with craniosynostosis. Untreated ICH causes developmental problems and may lead to loss of vision. Therefore, early detection and treatment of ICH is necessary.^1–3^

The best technique for diagnosing ICH in craniosynostosis is 24-hour invasive intracranial pressure (ICP) monitoring.^4^ However, this investigation requires a surgical procedure, thereby making it unfeasible for routine screening. Alternative methods for detecting ICH include evaluating the presence of risk factors for and indirect indicators of ICH. Risk factors include craniocerebral disproportion, hydrocephalus, obstructive sleep apnea, and cerebral venous hypertension.^5–7^ Indirect indicators include symptoms such as morning headaches and behavioral changes, fingerprinting on skull radiographs,^8–11^ and papilledema on fundoscopy. The assessment of fingerprinting is observer-dependent. There are also problems with the diagnostic accuracy of what may appear to be “papilledema.”^4,9–14^ For example, the presence of high hyperopia (HH) may interfere with the detection of papilledema, resulting in a mimic (ie, pseudopapilledema). However, the distinction between papilledema and pseudopapilledema may be evident on fundoscopy and by using optical coherence tomography (OCT) images of the fundus.^15–17^ In fact, recent studies in isolated craniosynostosis show a high correlation between the presence of papilledema on fundoscopy and OCT scans.^15–19^

Therefore, we aimed to evaluate the following in children with craniosynostosis: the diagnostic accuracy of fundoscopy and OCT in detecting ICH; the time course of retinal thickness after the treatment of ICH; and whether the presence of HH interfered with fundoscopy or OCT findings of papilledema.

## PATIENTS AND METHODS

All patients with syndromic, multisuture, unicoronal, unilambdoid, or sagittal synostosis who were managed between 2016 and 2020 and who were able to comply with OCT scanning were prospectively included. We also added patients who underwent OCT scanning before 2016 because of a clinical suspicion of ICH. All patients underwent cranial vault expansion in the first year of life, except for patients with a late referral.^6^ Patients with a late referral only underwent surgery when signs of ICH were present, or in case of severe distortion of the skull shape. The Erasmus Medical Center’s institutional review board approved this study (approval numbers MEC-2015-638 and MEC-2017-1143).

### OCT Protocol

The Spectralis OCT scanner (Heidelberg Engineering; Heyex software v6.7.13) was used to obtain OCT scans. For each OCT scan, 2 measures were evaluated: total retinal thickness (TRT) and OCT fundus imaging. The image of the optic nerve head (ONH) was evaluated by an experienced ophthalmologist (S.E.L.). Papilledema was defined as 360 degrees of edema of the optic disc or blurring of the optic disc margins with obscuration of blood vessels. To measure the TRT of the ONH, the ONH was centered, after which the retinal image was focused to optimize scan quality.^19,20^ An automatic segmentation algorithm included in the Heyex software detected the TRT reference layers (ie, inner limiting membrane and Bruch membrane). A circular chart with concentric rings at 1, 2, and 3 mm was positioned over the ONH, dividing the ONH into 8 equal quadrants. The mean thickness of the TRT was calculated with a precision of 0.2 µm. OCT scans in which less than 75% of the areas was available were excluded. According to a previous study using the same OCT device on children (age 4 to 10 years), a TRT greater than 503.6 µm was considered as increased, and thus as papilledema.^19^ Because no TRT normative values exist for the Spectralis device in adults, the adults were scored according to these values as well.

### Fundoscopy

Results of all fundoscopies performed during follow-up were extracted from the medical records. Fundoscopies were scored by the experienced ophthalmologist (S.E.L.) as either “papilledema” or “no papilledema.” Presence of HH or optic disc drusen was considered with the interpretation of the fundoscopy.

Patients underwent fundoscopy regularly according to our follow-up protocol:

- Sagittal synostosis: preoperatively and yearly from age 2 to 6 years
- Unicoronal and unilambdoid synostosis: preoperatively and at age 2, 3, and 4 years
- Crouzon syndrome: preoperatively, every 3 months from age 1 to 2 years, biannually from age 2 to 5 years, and annually from age 5 to 10 years
- Apert and Saethre-Chotzen syndrome and multisuture craniosynostosis involving the lambdoid sutures: preoperatively, biannually from age 1 to 5 years, and annually from age 5 to 10 years
- Muenke syndrome, complex craniosynostosis without lambdoid synostosis, *IL11RA* and *HUWE1* gene mutation craniosynostosis: preoperatively, biannually from age 1 to 2 years, and annually from age 2 to 10 years

Irrespective of age, additional fundoscopy was performed when ICH was suspected.

### Refractive Error

The spheric cycloplegic refractive error was measured by an experienced orthoptist and expressed in diopters (D). HH was defined as a spheric equivalent of greater than +4 D.

### Intracranial Hypertension

ICH was scored as being absent or present, determined by the following criteria:

- Invasive 24-hour ICP monitoring, with baseline findings classified as follows: less than 10 mm Hg, normal; 10 to 15 mm Hg, borderline abnormal depending on the height and duration of abnormal plateau waves (see following); greater than 15 mm Hg, abnormal. Trends in ICP values were also checked for any increase overnight. Abnormal ICP plateau waves were categorized as follows: plateau height less than 25 mm Hg (normal), 25 to 35 mm Hg (borderline), or greater than 35 mm Hg (abnormal), and plateau duration as less than 10 minutes (normal), 10 to 20 minutes (borderline), or greater than 20 minutes (abnormal).^21,22^
- Progressive ventriculomegaly (ie, hydrocephalus) and obliterated subarachnoid spaces on cerebral magnetic resonance imaging (MRI) scans.
- Progressive cerebellar tonsillar herniation (or syrinx) and obliterated subarachnoid spaces on cerebral MRI scans.
- Progressive fingerprinting on skull radiographs or scalloping of the inner cranial cortex on computed tomography scans, and reduced size of the ventricles or obliterated subarachnoid spaces.
- Occipitofrontal head circumference growth curve deflection, indicating craniocerebral disproportion.^6^

Presence of ICH was determined in 1 of 3 ways: if criterion A was abnormal, if criterion B or C was abnormal, or if criterion D and E were abnormal. Because the diagnostic accuracy of papilledema was evaluated, papilledema itself was not taken into consideration for the evaluation of the presence of ICH.

### Statistical Analysis

Statistical analyses were performed using R statistical software (version 4.0.3) and consisted of 2 major parts: diagnostic accuracy (primary outcome) and longitudinal follow-up (secondary outcome). The interference of HH was evaluated as a tertiary outcome. Statistical significance was set at a *P* value less than 0.05.

#### Diagnostic Accuracy

To examine the sensitivity, specificity, positive predictive value, negative predictive value (NPV), positive likelihood ratio, and negative likelihood ratio for fundoscopy findings, OCT fundus imaging, and TRT to detect ICH, we used data collected from the eye with the thickest TRT. In patients who developed ICH, the OCT scan and fundoscopy at the first moment that ICH was detected were used. In patients who had never had ICH who also had identical results for each scan or fundoscopy (ie, all were true negative or false positive), the first scan or fundoscopy was selected for analyses. In patients with contrasting results (ie, true negative results in 1 scan or fundoscopy and false-positive results in the other), the false-positive scans and fundoscopies were selected.

The sensitivity, specificity, positive predictive value, NPV, positive likelihood ratio, and negative likelihood ratios were calculated (contingency tables package version 2.0.0).

The odds ratios (ORs) of abnormal fundoscopy, OCT fundus imaging, or TRT results to detect ICH were analyzed by calculating ORs from generalized estimation equation models, using the cutoff value from normative values and correcting for age as a covariate and multiple measurements (geepack package version 1.3-1).^19^ For this analysis, all OCT scans were included up until the first moment that ICH was detected. The necessity of interaction terms was tested by evaluation of the quasi-likelihood under independence model criterion with and without interaction terms, and interactions were subsequently not entered into the models.

#### Longitudinal Follow-Up

The eye with the thickest TRT on the first OCT scan was selected for all consecutive measurements. Longitudinal time course of retinal thickness after the treatment of ICH was evaluated with 2 linear mixed models (nlme package version 3.1-150). Effect plots were generated with the ggplot2 package, version 3.3.2. We used 3 statistical models to examine these data. In the first model, all OCT scans in all patients who underwent surgery were included. TRT was defined as a continuous outcome variable, whereas a historical finding of ICH (yes/no) and age (continuous) were entered as independent predictors. The necessity of an interaction term between ICH and age was tested by evaluation of the Akaike information criterion with and without interaction terms, and an interaction was subsequently entered into the first model. In the second model, all patients who had had papilledema and ICH were included. TRT was defined as a continuous outcome; time after surgery in years was included as a continuous independent variable. In the third model, all patients were included. TRT in patients who did not have ICH but underwent surgery according to our protocol was compared with TRT in patients who did not undergo surgery because of late referral and no signs of ICH. In the statistical model, TRT was defined as a continuous outcome variable, whereas surgery (yes/no, but no ICH before surgery) and age (continuous) were the independent predictors. Again, the necessity of an interaction term between surgery and age was tested by evaluation of the Akaike information criterion with and without interaction terms, and an interaction was subsequently entered into the first model.

#### Interference of HH

The interference of HH on fundoscopy and OCT results was analyzed by calculating ORs from generalized estimation equation models. For this analysis, all OCT scans were included up until the first moment that ICH was detected. We used 3 models. In these models, papilledema on fundoscopy (1) or OCT fundus imaging (2) and increased TRT (3) were defined as a binary outcome (yes/no), and high HH (yes/no) and ICH (yes/no) were the independent predictors.

## RESULTS

**Fig. 1.**
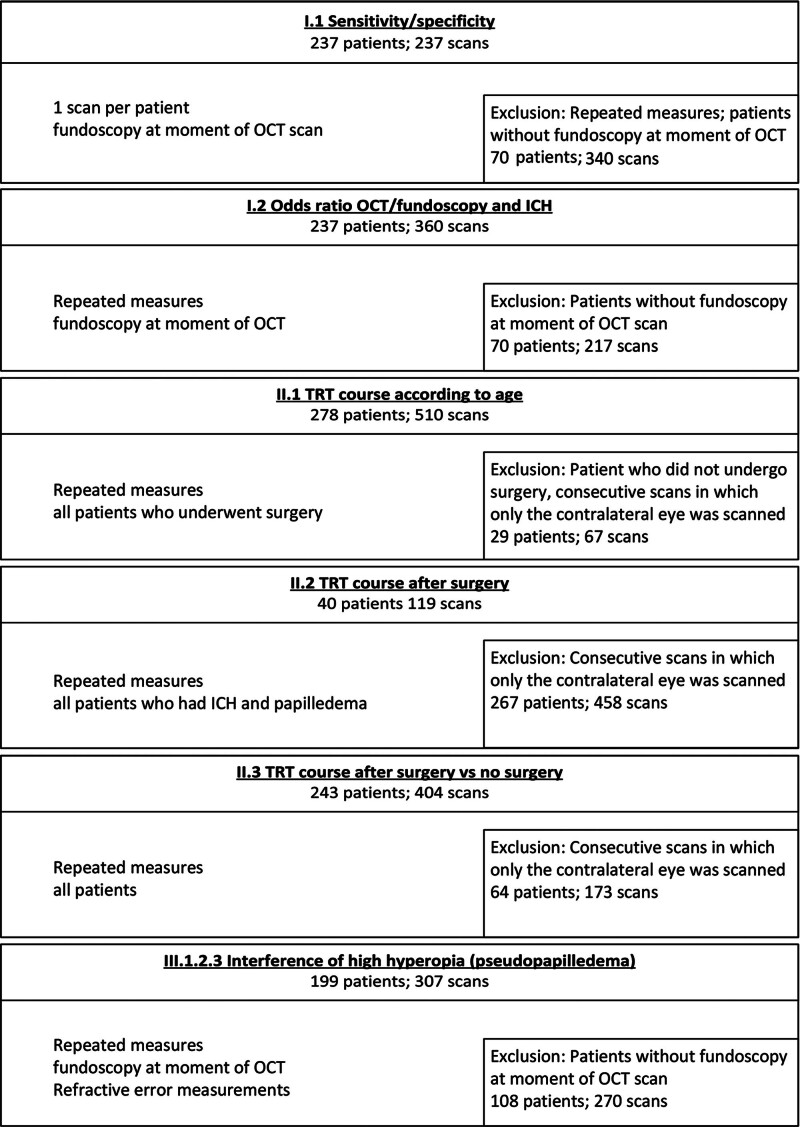
Patient inclusion in the analyses. Sections are not mutually exclusive.

The total study cohort included 307 patients (46% female) (Table 1). An attempt to perform an OCT scan failed in 5 patients: 3 had Apert syndrome, 1 had isolated unicoronal synostosis, and 1 had multisuture synostosis. Reasons to not attempt OCT scanning in children included a lack of focus, intellectual disability, or a (time-related) lack of parental consent. A total of 577 OCT scans were carried out, and the median age at OCT scan was 7.7 years (interquartile range [IQR], 5.5 to 11.2). Figure 1 depicts the inclusion and exclusion criteria per individual analysis (ie, diagnostic accuracy as the primary outcome, longitudinal follow-up as the secondary outcome, and the interference of HH as a tertiary outcome) and the numbers of included scans and patients per analysis. A total of 283 of 307 patients (92.2%) underwent surgery, and the median age at first surgery was 0.7 years (IQR, 0.5 to 1.0). Twenty-four patients did not have ICH and did not undergo surgery, 22 because of late referral and 2 because of parental choice. A subset of 131 of 307 patients (42.7%) underwent repeated OCT scanning, with a median number of 3 scans in these patients (IQR, 2 to 4; range, 2 to 11), and a median follow-up period between the first and last OCT of 2.1 years in this specific subset of patients (IQR, 1.0 to 4.4; range, 0.1 to 9.8). Manual corrections of the segmentation lines for the TRT were performed in 69 of 577 OCT scans (12%).

**Table 1.** Patient Characteristics

Diagnosis	No. of Patients
Syndromic craniosynostosis, *n*	
Apert	16 (1 unoperated)
Crouzon-Pfeiffer	43 (7 unoperated)
Muenke	22 (1 unoperated)
Saethre-Chotzen	19 (1 unoperated)
Multisuture craniosynostosis	29 (3 unoperated)
*IL11RA* craniosynostosis	2
*HUWE1* craniosynostosis	1
Isolated craniosynostosis, *n*	
Unicoronal synostosis	28 (2 unoperated)
Sagittal synostosis	138 (9 unoperated)
Lambdoid synostosis	9
Female sex, *n* (%)	140 (46)
Age at first surgery, yrs, median (IQR)	0.7 (0.5–1.0)
Age at OCT scan, yrs, median (IQR)^a^	7.7 (5.5–11.2)
No. of OCT scans, median (IQR)^b^	3 (2–4)
Follow-up time between first and last OCT scan, yrs, median (IQR)^b^	2.1 (1.0–4.4)

a Median age at all 577 OCT scans (thus including multiple measurements).

b In patients who underwent multiple OCT measurements (131 of the 307 patients).

### Diagnostic Accuracy

#### Sensitivity and Specificity of OCT and Fundoscopy

A total of 237 patients were included (Fig. 1); 17 (7.2%) had ICH during the fundoscopy and OCT scan (Table 2). Of these 17 patients, 9 had syndromic craniosynostosis (Crouzon, *n* = 7; Saethre-Chotzen, *n* = 1; *IL11RA*, *n* = 1), 1 had multisuture synostosis, and 7 had isolated craniosynostosis (sagittal, *n* = 6; unicoronal, *n* = 1).

**Table 2.** Classification of Intracranial Hypertension

Diagnostic Evaluation of Intracranial Hypertension	No. of Patients
Criterion A: Elevated intracranial pressure on 24-hour monitoring	8
Criterion B: Progressive ventriculomegaly and obliterated subarachnoid spaces	4^a^
Criterion C: Progressive cerebellar tonsillar herniation and obliterated subarachnoid spaces	4^a^
Criteria D and E: Progressive scalloping of the inner cranial cortex and reduced size of ventricles or obliterated subarachnoid spaces, occipital frontal head circumference growth curve deflection	3

a Two patients were classified by both criteria B and C.

An increased TRT was diagnosed in 43 of 237 patients (18.1%). OCT fundus imaging results were abnormal in 31 of 237, and fundoscopy results in 29 of 237. Diagnostic accuracy was evaluated for the TRT, OCT fundus imaging, and fundoscopy separately, for all 3 combined, and for TRT and OCT fundus imaging combined (Table 3). In this analysis, the pretest probability of signs of ICH was 7.2% (~1 in 14), and the posttest probability of ICH using all 3 diagnostic evaluations combined was 23.8% (~1 in 4).

**Table 3.** Diagnostic Accuracy of Total Retinal Thickness, OCT Fundus Imaging, and Fundoscopy to Detect Intracranial Hypertension

Test	Sensitivity^a^	Specificity^a^	Positive Predictive Value^a^	Negative Predictive Value^a^	Positive Likelihood Ratio	Negative Likelihood Ratio	Pretest and Posttest Probability for ICH, %
TRT, %	71 (12/17) (64–76)	86 (189/220) (81–90)	28 (12/43) (23–34)	97 (189/194) (95–99)	5.0 (3.0–7.8)	0.3 (0.12–0.62)	7.2 → 28.6
OCT fundus imaging, %	59 (10/17) (52–65)	90 (199/220) (86–94)	32 (10/31) (27–38)	97 (199/206) (93–98)	6.2 (3.2–11.0)	0.5 (0.2–0.7)	7.2 → 31.5
Fundoscopy, %	59 (10/17) (52–65)	91 (201/220) (87–94)	34 (10/29) (29–41)	97 (201/208) (93–98)	8.2 (4.7–14.3)	0.3 (0.1–0.6)	7.2 → 34
TRT + OCT fundus imaging, %	71 (12/17) (64–76)	83 (183/220) (78–87)	24 (12/49) (19–30)	97 (183/188) (94–99)	4.2 (2.6–6.3)	0.4 (0.1–0.6)	7.2 → 25
TRT + OCT fundus imaging + fundoscopy, %	76 (13/17) (71–81)	81 (179/220) (76–86)	24 (13/54) (19–30)	98 (179/183) (95–99)	4.1 (2.7–5.9)	0.3 (0.1–0.6)	7.2 → 24

a Values are absolute numbers (95% CI).

#### Odds Ratios of OCT and Fundoscopy

A total of 360 OCT scans in 237 patients were analyzed. There was a 13-fold or 15-fold greater odds of having ICH when TRT was increased, or papilledema was present on fundoscopy, respectively (Table 4).

**Table 4.** Odds of Having Intracranial Hypertension in Patients with Increased TRT or Papilledema on OCT Fundus Imaging or Fundoscopy (Dichotomous Outcomes)

Odds of Having Intracranial Hypertension	OR (95% CI)	*P*
Increased TRT	13.2 (4.3–40.5)	<0.001
Papilledema on OCT fundus imaging	12.3 (4.2–35.7)	<0.001
Papilledema on fundoscopy	15.0 (5.0–45.1)	<0.001
Increased TRT or papilledema on OCT fundus imaging or papilledema on fundoscopy^a^	11.9 (3.7–37.6)	<0.001
Increased TRT and papilledema on OCT fundus imaging^b^	9.6 (3.3–28.2)	<0.001

a Odds of having ICH if 1 of the following indicated ICH: TRT, OCT fundus imaging, or fundoscopy.

b Odds of having ICH if 1 of the following indicated ICH: TRT or OCT fundus imaging.

### Longitudinal Follow-Up of TRT after the Treatment of ICH

#### Course of TRT by Age in Patients Who Underwent Surgery

**Fig. 2.**
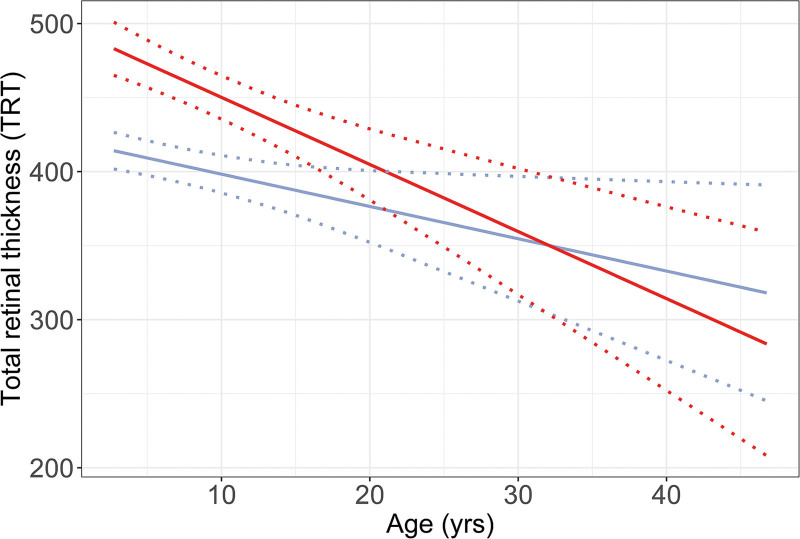
Effect plot of linear mixed model, visualizing the course of total retinal thickness in patients who had had ICH (*red line*) versus patients who had never had ICH (*blue line*).

A total of 510 OCT scans were carried out in 278 patients (Fig. 1). A total of 56 patients had a history of ICH. TRT was increased in patients who had had ICH versus patients who had never had ICH (β +44.9 µm in TRT [95% CI, 9.0 to 80.8]). Moreover, the TRT slightly decreased as patients aged (β −4.0 µm/yr [95% CI, −5.8 to −2.2]) (Fig. 2). The observed decrease by age was not different in patients who had had ICH versus patients who had never had ICH (β +0.4 [95% CI, −2.7 to 3.5]).

#### Course of TRT after Surgery in Patients Who Had Had Papilledema and ICH

A total of 119 scans in 40 patients were used, including only patients who had had papilledema and ICH (Fig. 1). These included 18 of 119 measurements performed after surgery according to protocol, 77 of 119 after repeated surgery because of recurrent ICH, and 24 of 119 because of ICH in patients with a late referral. TRT was significantly decreased in the years after surgery (β −3.6 µm/yr [95% CI, −7.2 to −0.05]).

#### Course of TRT in Patients Who Did Not Undergo Surgery versus Patients Who Underwent Surgery but Never Had ICH

**Fig. 3.**
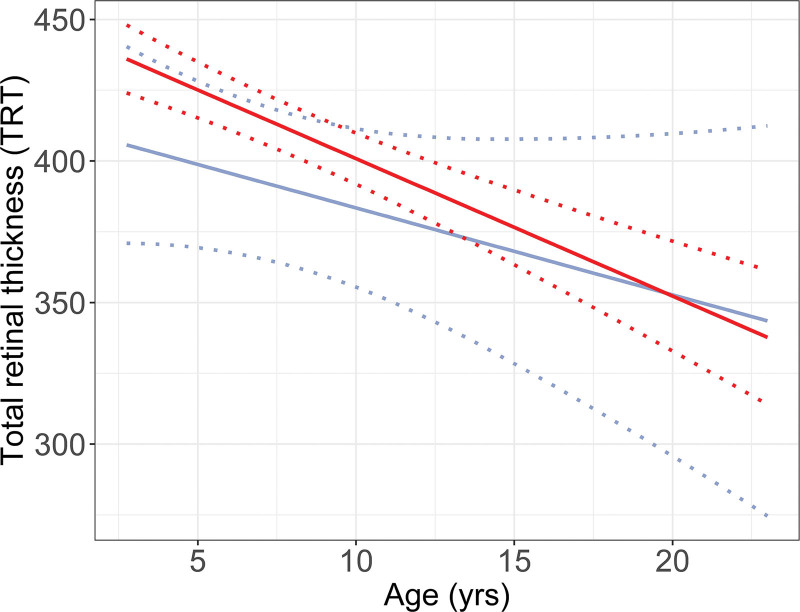
Effect plot of linear mixed model, visualizing the course of total retinal thickness in patients who never underwent surgery (*blue line*) versus patients who underwent surgery, but never had ICH (*red line*).

In this analysis, 412 scans were carried out in 250 patients (Fig. 1). TRT in the 24 patients who did not undergo surgery was comparable to TRT in patients who underwent surgery according to the protocol, but never had ICH (β +35.2 µm [95% CI, −10.6 to 81.1]) (Fig. 3).

### Interference of HH on Fundoscopy and TRT

We analyzed 307 OCT scans carried out in 199 patients (Fig. 1). In total, 46 of 199 (23.1%) had HH. There was close to 3-fold greater odds of having an increased TRT if HH was present, corrected for the true presence of ICH (OR, 2.9 [95% CI, 1.4 to 7.6]). In contrast, there did not appear to be greater odds of having papilledema on OCT fundus imaging or fundoscopy in patients with HH, corrected for the true presence of ICH (OR, 2.3 [95% CI, 0.8 to 6.7] and OR, 3.1 [95% CI, 0.9 to 9.8]).

## DISCUSSION

Our study provides 3 key observations about quantitative detection and follow-up of ICH in patients with craniosynostosis.

### Diagnostic Accuracy

Following a sensitivity of 76% for the combination of OCT and fundoscopy, approximately 24% of patients do not develop papilledema or increased retinal thickness in the presence of signs of ICH (false-negative measurements).

The sensitivity of 59% for fundoscopy alone is remarkably higher compared with the 11% to 22% reported in the literature.^10,16^ In contrast, the sensitivity on OCT measures seems to be slightly lower compared with the numbers in the study by Swanson et al.^16^ Explanations for this difference include the alternative method to diagnose ICH (ie, the ICP was measured under general anesthesia and for 1 minute), and the other OCT measures used to evaluate retinal thickness. The difference between the fundoscopy sensitivity reported by Tuite et al.^10^ and the current study includes the different patient population (ie, the majority of their population had nonoperated isolated craniosynostosis) and the alternative method to assess ICP (ie, only baseline ICP was evaluated). Besides, the recorded time of the ICP was only 3 hours in some patients, whereas the recommended standard is a 24-hour overnight measurement.^4^

### Longitudinal Follow-Up

This study shows that TRT slightly decreases as children age, and this was comparable in children who had had ICH versus children who had never had ICH. Patients who had had ICH retained a significantly increased TRT compared with patients who had never had ICH, which is consistent with a previous study from our group.^20^ After surgery, TRT quickly decreases below the upper limit of normal values.^19^ We hypothesize that in some patients the retinal thickness remains in the upper bound of normal because of permanent anatomic changes.^23^

We did not identify a difference in TRT in late referred patients without ICH who never underwent surgery and patients who underwent surgery according to the treatment protocol, but who had never had ICH. This finding most likely reflects a mild phenotype in patients with a late referral. It appears that a conservative approach to surgical intervention in this particular group is safe, although long-term assessment of cognition and psychosocial well-being should be undertaken.

### Influence of HH

HH did not influence the OCT fundus imaging or fundoscopy outcomes; only TRT was influenced by this refractive anomaly. HH is commonly associated with a crowded optic nerve head, which causes pseudopapilledema.^24^ The association between an increased TRT and HH reflects both a strength and weakness of TRT, as the precision of the measurement up to a few microns likely results in earlier detection of retinal changes, but it also causes more false-positive measurements. These findings represent an additional argument to combine the OCT scan with fundoscopy. Also, total retinal volume might be of added value to distinguish papilledema from pseudopapilledema in patients with an increased TRT.^25^ Caution with the interpretation of this tertiary analysis is warranted; the CIs of the OR were wide.

### Clinical Diagnosis of ICH

The practice in our center is to check for signs of ICH at every visit, besides fundoscopy and OCT, including complaints of headaches in the morning, changes in behavior or sleep pattern, and decline of occipitofrontal head circumference. If ICH is suspected, we proceed with imaging (computed tomography or MRI) and, sometimes, invasive ICP measurement. The current study underlines the importance of additional diagnostic aids for accurately screening for ICH. Although the pre- to posttest increase in probability of ICH being present was modest (7.2 to 24%, or 1 in 14 to 1 in 4) for certainty in identifying ICH, it is better than using clinical assessment alone. In patients with craniosynostosis, a high sensitivity and NPV are essential to prevent consequences of prolonged ICH.^1–3,26^

Our study has limitations. First, only 17 patients were diagnosed with ICH, which resulted in pretest probability for signs of ICH of only 7.2% and limited the evaluation of sensitivity. This low number may be related to our protocol with routine surgery within the first year of life. Also, we only had invasive ICP measurements in 8 patients, and the majority of the cases with ICH were diagnosed based on proxy measurements. Invasive measurements represent the best technique for diagnosing ICH in craniosynostosis, but are unfeasible for routine screening, as a surgical procedure and inpatient care for 24 hours are required. The calculated sensitivity and specificity should therefore be interpreted with caution. However, we used several other clinical derivatives of and risk factors for ICH to evaluate the presence of ICH, such as ventriculomegaly, Chiari I malformation, and skull growth arrest. These individual risk factors potentially suffer from measurement error, but pooling them together reduces the chance of missing or overdiagnosing ICH. Also, we analyzed the diagnostic accuracy based on a group of patients with isolated and syndromic craniosynostosis who are at risk of developing ICH. This may have influenced our calculated measures, as the risk of ICH varies within these diagnoses. For future studies, we would recommend an analysis per diagnosis, in patients with a low and high risk of developing ICH, within a greater sample size.

We did not have enough data to analyze the diagnostic accuracy and longitudinal follow-up of the peripapillary retinal nerve fiber layer, an OCT measure which has been studied by others.^16,18^ However, the TRT and TRV are apparently more accurate in monitoring and diagnosing papilledema or ICH, especially in the pediatric age group.^27–29^

## CONCLUSIONS

The sensitivity of TRT, OCT fundus imaging, and fundoscopy to detect the presence of signs of ICH is 71%, 59%, and 59%, respectively, and 76% when the 3 methods are combined. We found that the presence of HH affects the OCT findings and needs to be considered when evaluating OCT results. An increased TRT (after excluding HH) and papilledema on fundoscopy, combined with a clinical suspicion of ICH, warrants further screening for the presence of ICH. During follow-up after surgery, the increased TRT due to ICH remains higher compared with the TRT of age-matched patients without ICH, but falls within the bounds of normative values.

## DISCLOSURE

The authors have no financial interests to disclose.

## ACKNOWLEDGMENTS

This study was funded by the Stichting Lijf en Leven foundation (Krimpen aan de IJssel, the Netherlands, grant no. 16-155). The authors would like to thank Stichting Lijf en Leven for their financial contribution, which has made this work possible. They would also like to thank their experienced ophthalmologists Ruben de Kok and Gabriëlle Buitendijk and pediatric optometrists Marieke Telleman and Rhodé Bregman.
